# Gender Stereotypes and Peer Selection in STEM Domains Among Children and Adolescents

**DOI:** 10.1007/s11199-022-01327-9

**Published:** 2022-11-04

**Authors:** Luke McGuire, Adam J. Hoffman, Kelly Lynn Mulvey, Adam Hartstone-Rose, Mark Winterbottom, Angelina Joy, Fidelia Law, Frances Balkwill, Karen P. Burns, Laurence Butler, Marc Drews, Grace Fields, Hannah Smith, Adam Rutland

**Affiliations:** 1 Department of Psychology Washington Singer Building, University of Exeter, Exeter, Perry Road, Exeter EX4 4QG, United Kingdom; 2 North Carolina State University, Raleigh, United States; 3 University of Cambridge, Cambridge, United Kingdom; 4 Centre of the Cell, Queen Mary University of London, London, United Kingdom; 5 Virginia Aquarium & Marine Science Center, Virginia Beach, United States; 6 Thinktank Science Museum, Birmingham, United Kingdom; 7 EdVenture, California, United States; 8 Riverbanks Zoo & Garden, Columbia, United States; 9 The Florence Nightingale Museum, London, United Kingdom

**Keywords:** STEM stereotypes, Gender stereotypes, Peer selection, Social identity

## Abstract

Gender stereotypes are harmful for girls’ enrollment and performance in science and mathematics. So far, less is known about children’s and adolescents’ stereotypes regarding technology and engineering. In the current study, participants’ (*N* = 1,206, girls *n* = 623; 5–17-years-old, *M* = 8.63, *SD* = 2.81) gender stereotypes for each of the STEM (science, technology, engineering, and mathematics) domains were assessed along with the relation between these stereotypes and a peer selection task in a STEM context. Participants reported beliefs that boys are usually more skilled than are girls in the domains of engineering and technology; however, participants did not report gender differences in ability/performance in science and mathematics. Responses to the stereotype measures in favor of one’s in-group were greater for younger participants than older participants for both boys and girls. Perceptions that boys are usually better than girls at science were related to a greater likelihood of selecting a boy for help with a science question. These findings document the importance of domain specificity, even within STEM, in attempts to measure and challenge gender stereotypes in childhood and adolescence.

Within science, technology, engineering, and mathematics (STEM) fields, women are almost reaching parity of representation in some areas but remain underrepresented in others. For example, in the physical sciences (physics, chemistry, and related subjects) women represent 43% of graduates (WISE, 2020b) and 46% of science professionals in the United Kingdom (UK) (WISE, 2021). In the United States (US), women earn 59% of biology undergraduate degrees. In stark contrast, in the UK, women represent 14% of the computer science cohort in secondary education, 16% of computer science graduates, and 17% of computer science professionals (WISE, 2020a, 2020b, 2021). Similarly, in engineering, women represent 16% of graduates and 10% of UK engineering professionals (WISE, 2020b, 2021). In the US women earn fewer than 20% of computer science and engineering degrees (National Science Foundation, 2018). Stereotypes about who *can* and *should* succeed in these domains have been shown to be related to motivation, interest, and achievement (Mulvey et al., 2022), which are in turn crucial for the uptake of these subjects.

Gender stereotypes are strongly related to STEM interest, motivation and performance in childhood, adolescence, and adulthood (Bleeker & Jacobs, 2004; Evans et al., 2011; Mulvey & Irvin, 2018; Schuster & Martiny, 2017; Starr, 2018; Swinton et al., 2011). Much of the research examining STEM gender stereotypes in childhood and adolescence has focused specifically on science and math (e.g., Cvencek et al., 2011; Ertl et al., 2017). Despite a burgeoning focus on computer science interest (Master et al., 2016, 2021), there is a need for research that systematically examines and compares youth’s gender stereotypes in the *four* STEM domains across different age groups. The present study extends the developmental literature by examining domain-specific gender stereotypes about ability in youth ranging in age from 5- to 17-years-old. Further, we examine one possible consequence of such stereotypes by exploring how these stereotypes are related to the gendered choices made by young people when choosing peers to help them in a STEM context. The current work was guided by a social identity theoretical framework, which emphasizes the importance of stereotypes in guiding attitudes and behavior, having formed through the emergence of categorical thinking based on social identities.

## Social Identity Framework

The social identity approach, drawing on social identity theory (Tajfel & Turner, 1986) and self-categorization theory (Turner et al., 1987), has proposed that individuals categorize themselves and others along a number of salient identities into in-groups (those groups we identify with and seek to belong to) and out-groups (other groups we do not belong to). Research drawing from social identity development theory (SIDT; Nesdale 2007) has documented that these in-group preferences emerge in childhood (around 5–8-years-old) where children also demonstrate a concern for promoting and maintaining positive aspects of their group identity. This categorization process contributes to the formation of stereotypes: shared knowledge structures about social groups that may or may not accurately reflect characteristics of those groups (Devine, 1989).

Gender is a central identity that children use to understand and categorize their social world (Liben & Bigler, 2002; Shutts et al., 2013). Stereotypes about different gender groups (e.g., their roles, behaviors, appearance) solidify shortly after children begin to understand gender labels (Ruble et al., 2006) and alongside their emerging awareness of their membership of social identity groups (Nesdale & Flesser, 2001). Children may vary in their understanding of the stability of gender identity across development, yet research indicates that gender stereotypes tend to be endorsed more strongly by young children who are learning that gender stays constant across time (i.e., gender stability) (O’Keefe & Hyde, 1983; Ruble et al., 2007). It is important to note that contemporary scholarship has moved away from gender binary conceptualizations of gender identity (see Hyde et al., 2019), yet much of the social world continues to be structured in such a way that children are encouraged to think about gender as a binary social category.

This early development of gender stability means young children are motivated to attend to, learn about, and adhere to gender roles, thus contributing to increased gender stereotyping. Following the emergence of gender stability in early childhood (3–5 years), children between 5 and 7 years begin to understand gender consistency, or the idea that gender remains consistent across situations even if superficial elements (e.g., hair styles) change. This emerging understanding that gender is not tied to showing or believing in stereotypic features (e.g., ‘all girls should have long hair’) leads to an accompanying decrease in rigid gender stereotypes. Alongside this, the emergence of multiple classification abilities between 7 and 11-years-old (Bigler & Liben, 1992) leads to further declines in rigid gender stereotyping. This ongoing cognitive development would suggest that, in terms of STEM gender stereotyping, younger children would endorse stereotypes about boys’ abilities that would then decline with age.

Importantly, cognitive development also occurs alongside social development and knowledge. From a social developmental perspective, the status of one’s in-group comes to play an important role in the endorsement of stereotypes. In childhood (between 5 and 8-years-old, around the same time that children begin to develop a preference for their in-groups within the SIDT framework), children report in-group favoritism in their math stereotypes (Heyman & Legare, 2004; Passolunghi et al., 2014) and on a generalized STEM stereotype measure (McGuire, Mulvey, et al., 2020). With age and emerging understanding of status differences, members of higher status groups are more likely than those from lower status groups to show an in-group bias, a pattern which has been demonstrated using minimal group paradigms with children (Bigler et al., 2001). In contrast, members of lower status groups are more likely to endorse favorable stereotypes about higher status groups in areas where these status differences are apparent. This pattern of results has been documented in relation to children’s perceptions of academic ability, with higher status groups (i.e., boys) reporting stereotypes that favor their in-group (Rowley et al., 2007).

Although stereotypes may be cognitive efficient when navigating a complex social world, they have powerful consequences for reinforcing ideas about who *can* or *should* succeed in particular domains (Master & Meltzoff, 2020), which in turn can impact performance and motivation through processes including (but not limited to) stereotype threat (see Spencer et al., 2016 for a review of stereotype threat literature, and Shapiro & Williams 2012, for an example in the context of STEM). In line with the tenets of the social identity approach, we may expect that early endorsement of in-group abilities would give way to the endorsement of stereotypes that favor the abilities of higher status groups. In the case of STEM, this would involve children initially endorsing the abilities of their own gender group, before endorsing boys’ abilities later in childhood. This ability endorsement may be greater still in the engineering and technology domains where gender disparities are most apparent.

## STEM Gender Stereotypes Across Childhood and Adolescence

In line with the expectations of the social identity approach, recent research has documented an in-group bias among boys between 5- and 8-years-old when asked “who is usually good at STEM” (McGuire, Mulvey, et al., 2020). This in-group preference was greater in middle childhood than in adolescence. In contrast, girls were less likely to report in-group favoring responses to this stereotype measure in early childhood, with this also being the case in middle childhood and adolescence. One possibility then is that status differences in STEM are so immediately apparent that girls do not promote a positive image of their own group. However, McGuire et al., (2020) examined a mean score that represented children’s endorsement of gender stereotypes about all *four* STEM subjects combined. One alternative possibility, unexamined by previous work, is that children and adolescents may hold different stereotypes about the four different STEM domains where relative status differences between groups may be smaller (e.g., life sciences) or larger (e.g., engineering). To explore this, the present study offers the first examination and comparison of age-related differences in gender stereotypes across science, technology, engineering, and mathematics.

Although there has yet to be a single study that examines children’s and adolescents’ gender stereotypes in the four domains of STEM simultaneously, research has examined stereotypes in these domains independently, with a particular focus on math. Stereotype findings from the math domain are mixed. Some researchers have demonstrated, using implicit and explicit measures, that even children as young as 6-years-old associate math with boys more than with girls, with others showing similar findings across development into adulthood (Cvencek et al., 2011; Dweck, 2007; Guiso et al., 2008; Lummis & Stevenson, 1990; Nosek et al., 2009). However, other evidence has shown that youth over 10 years old either report that they do not believe there to be gender differences in mathematics ability (Kurtz-Costes et al., 2008; Martinot et al., 2012; Muzzatti & Agnoli, 2007; Plante et al., 2009; Rowley et al., 2007) or instead favor girls’ math ability (Kurtz-Costes et al., 2008; Martinot & Désert, 2007). This latter evidence may represent a shift in status differences within math, as evidence from the last decade documents a decline in gender differences in measures of math ability (Lindberg et al., 2010; O’Dea et al., 2018). The present study makes an important contribution to this body of literature by examining these math gender stereotypes in comparison with technology and engineering, where status-related gender differences are still clearly apparent. Comparing age-related differences in these stereotypes will provide evidence regarding the salience of these status differences for children and adolescents.

Stereotypes about engineering and technology have generally been measured among older youth samples. Adolescent girls have been shown to underestimate their own ability in computer science and engineering, which in turn is related to lower interest in participating in these fields (Correll, 2001; Ehrlinger & Dunning, 2003) and lower enrollment in high school computer programming classes and courses (Schumacher & Morahan-Martin, 2001; Weisgram & Bigler, 2006). Within the age range where decisions are beginning to be made about further study, it appears beliefs about superior male ability in engineering and technology are already being endorsed by adolescents.

Crucially, a study by Master et al., (2017) examined gender stereotypes about robotics and programming, as well as math and science, among 6-year-old boys and girls. Participants of both gender groups reported that boys were better than girls at robotics and programming but did not hold these same stereotypes about math and science. Importantly, these stereotypes can be culturally and contextually transmitted (Cheryan et al., 2015), which may help to explain why from a young age, girls may endorse these stereotypes even when they have not participated in technology or engineering classes at school. For example, women surveyed after sitting in computer science classrooms that include stereotypical cues (e.g., science-fiction film posters) report lower interest in computer science compared to women surveyed after sitting in classrooms with non-stereotypical cues (e.g., art posters; Cheryan et al., 2009). Similarly in adolescence (14–18-years), girls were more likely to opt to enroll in a computer science class when the class took place in a non-stereotypical classroom context (Master et al., 2016). Research has therefore documented that girls hold a belief that boys are better at computer science activities or do not see computer science as “for me” (Master et al., 2017). These findings fit with expectations derived from the social identity approach: where status differences are most apparent and communicated by our surroundings (e.g., Cheryan et al., 2009), members of lower status groups will endorse stereotypes favoring the abilities of higher status groups.

## Peer Selection in STEM

As well as extending the current literature on STEM gender stereotypes by examining domain-specific gender ability stereotypes across childhood and adolescence, the present study also examines whether these gender stereotypes are related to decisions about peer selection in a STEM context. Exclusion by peer group members has consequences for well-being and motivation in the school context (Killen & Rutland, 2011). Peer group norms, which are often founded upon stereotypical ideas, are related to decisions about who is included and who is excluded in a group situation. For example, starting in middle childhood, children understand that their peers are more likely to socially include someone who adheres to a conventional group norm and that those who challenge these norms will likely be ostracized (Killen et al., 2013). In the STEM context, children in middle childhood, especially boys, perceive that their peers will less positively evaluate someone who challenges a peer group norm for a gendered STEM activity (McGuire, Jefferys, et al., 2020). In this study, boys negatively evaluated an in-group peer who wanted to take part in a biology activity (which was the activity that a girls’ group wanted to do) when the rest of the boys’ group wanted to take part in a computer science activity. In contrast, girls did not negatively evaluate a peer who wanted to take part in a different STEM activity than the girls group. These findings align with the emerging importance of group identity for children along with the relative importance of maintaining status through these decisions. Specifically, compared to girls, boys perceive a greater threat to their status from a peer who challenges the group’s norm based on stereotypes about STEM.

Though researchers have shown that children take peer group norms into account when making decisions about peer evaluation and inclusion in STEM contexts, less is currently known about how STEM gender stereotypes are related to children’s decisions about the peers that they will seek STEM expertise from. In the present study we asked participants to choose a peer to ask for help with a difficult science question, with the opportunity to select between a male character and a female character of equal STEM interest and ability. Work with adults has shown that even when objective ability is matched, beliefs about male ability often leads to a bias towards male characters in fictional recruitment tasks (González et al., 2019). Such decisions can serve to strengthen and perpetuate ideas about ability, as those who are chosen to take positions of power or vocalize their knowledge may appear to confirm stereotypes about male ability. We expected that greater endorsement of male ability in a direct stereotype measure would be related to a greater likelihood of selecting a male character for help in a STEM task. This task is intended to serve as one representation of the potential consequences that early endorsement of STEM ability stereotypes may have for children and adolescents.

## The Current Study

The current study provides the first direct examination and comparison of gender stereotypes in each of the STEM domains across childhood and adolescence. Further, we extend the existing literature by examining how these stereotypes are related to peer selection in a STEM context. Participants between 5- and 17-years-old completed a survey including STEM stereotype measures and a STEM peer selection task measure.

## Hypotheses

**H1.** Stereotype endorsement for math and science in middle childhood (5–8-years-old) was expected to be stronger compared to late childhood (9–11-years-old) or adolescence (12–17-years-old), especially among boys. These age and gender differences were anticipated due to the emerging importance of maintaining a positive image of one’s in-group in middle childhood and increasing exposure in late childhood and adolescence to the relatively smaller gender differences in status and representation in math and science compared to engineering and technology.

**H2.** In contrast, stereotype endorsement for engineering and technology was expected to be stronger in late childhood and adolescence, compared to middle childhood, for both boys and girls. These age differences were expected due to the cultural transmission of ideas about male ability (i.e., status differences) in engineering and technology (Cheryan et al., 2015) and exposure to inequitable gender representation (i.e., representation differences) in these two domains increasing with age (WISE, 2020a, 2020b).

**H3a.** In a measure of gendered help-seeking among peers in a science task, we expected that selecting a male character would be predicted by (a) greater endorsement of stereotypes about male ability in science and (b) perceptions of the male character’s ability in science.

**H3b.** Further, we expected that boys (due to in-group bias) and younger participants (due to stronger science stereotypes among younger participants, as shown in extant work; McGuire et al., 2020) would be more likely (compared to girls and older participants, respectively) to select a male character to help them in a science task.

**H3c.** Along with testing for these main effects (stereotypes, character ability perceptions, participant gender, participant age), we tested the two-way interaction between participant age and gender. Here we expected that for girls, age would be negatively related to selecting the male character, given that stereotype endorsement for science becomes weaker with age due to cultural transmission of relatively smaller gender differences in status and representation in science (as per H1). This is further expected in part because, for boys, in-group bias, as a means to protect relatively higher in-group status, may disguise age-related differences (i.e., both younger and older boys would select the male character).

## Method

### Participants

Participants (*N* = 1,206, girls = 623, boys = 583) were recruited from six informal science learning sites (ISLS). These included a zoo (*n* = 194), an aquarium (*n* = 265), and a children’s science museum (*n* = 115) located in the Southeastern US, as well as a science museum (*n* = 483) in the Midlands of the UK, and a children’s biomedical science center (*n* = 75) and medical history museum (*n* = 74) in the Southeast of the UK. Participants were divided into three age groups: middle childhood (*n* = 650, *M*_age_ = 6.53, *SD* = 1.06, min. = 5-years-old, max. = 8-years-old), late childhood (*n* = 359, *M*_age_ = 9.91, *SD* = 0.82, min. = 9-years-old, max. = 11-years-old), and adolescence (*n* = 185, *M*_age_ = 13.57, *SD* = 1.63, min. = 12-years-old, max. = 17-years-old).

64% (*n* = 770) of participants identified as members of the ethnic majority group of the country of testing (White British in the UK sites [*n* = 420, 35.1%]), White or European American in the US sites [*n* = 350, 29.2%]). In the US, the sample also included Black/African American (*n* = 100, 8.3%), Bi-Racial/Multi-Racial (*n* = 29, 2.4%), Hispanic/Latinx (*n* = 32, 2.7%), Asian (*n* = 11, 0.9%), Pacific Islander (*n* = 10, 0.8%), and American Indian (*n* = 10, 0.8%) participants. In the UK, the sample also included Mixed Race/Dual-Heritage (*n* = 53, 4.4%), Indian British (*n* = 34, 2.8%), Black British (*n* = 23, 1.9%), Pakistani British (*n* = 21, 1.7%), Chinese British (*n* = 17, 1.4%), and Bengali British (*n* = 6, 0.5%) participants. There were 81 (6.7%) participants who indicated their ethnicity as Other, and 9 (0.7%) participants did not report their ethnicity. Parental consent and child assent were obtained for all participants in the UK and parental notification and child assent established for all participants in the US, as per IRB requirements.

## Procedure

All measures were approved by the North Carolina State University IRB and Goldsmiths, University of London Ethics Committee as part of the ‘STEM Teens’ project. The protocol was completed in the ISLS using either online survey software (Qualtrics, Provo, UT) on a tablet computer, or in hard copy. In both cases the same measures were utilized. Participants could select to complete the survey independently or in a one-to-one interview format with an experimenter. The survey took approximately 10 to 15 minutes to complete.

Participants were recruited on site by experimenters, and offered either an electronic gift card, gift shop voucher or gift bag worth $/£5 in exchange for completing a questionnaire. Participants were part of family groups visiting the site, consisting of at least one adult and one child. All participants were approached at the exit of pre-selected galleries or exhibits. These exhibits were chosen in conjunction with ISLS staff and recognized as popular areas of the ISLS.

## Measures

The below measures were part of a larger questionnaire that also included measures related to STEM learning, motivation, and engagement in ISLS.

## Gender Stereotype Measure

The gender stereotype measure utilized in the current study was adapted from Liben & Bigler (2002). To avoid participants being asked to directly compare the ability of boys and girls we adapted the original measure to assess perceptions of STEM ability separately for boys and girls. Participants were asked to read a sentence and then use a slider (or in the hard copy version, mark on a line) to indicate their agreement with the sentence. Participants answered four single-item questions about boys’ ability, and four questions about girls’ ability. The questions were as follows:

“I think that girls usually do well in [domain name]” (0 = *not true at all*, 100 = *very much true*, slider marked in increments of 10).

“I think that boys usually do well in [domain name]” (0 = *not true at all*, 100 = *very much true*, slider marked in increments of 10).

Participants responded to both questions for each STEM domain. To ensure our youngest participants understood what we meant by engineering and technology, we included examples of “technology means things like using computers” and “engineering means things like building machines”.

Using these items and given the tendency for stereotypes about STEM to favor boys, we created stereotype response difference scores representing each STEM domain (science, technology, engineering, mathematics) by subtracting the response to the question about girls from the response to the question about boys. These scores were scaled from − 100 (responded 100 to girls question and 0 to boys question = minimum stereotype endorsement) to + 100 (responded 100 to boys question and 0 to girls question = maximum stereotype endorsement).

## Peer Selection Task

### Participants were Asked to Read the Following Scenario:

“Imagine you are part of an after-school science group. The group likes to meet up to work together on different science projects for fun. You are working on a project together and there is a question you don’t know the answer to. There are two other people sitting at your table who you can ask for help. These people are…”.

Participants were then presented with two profiles (see Table 1). One profile was accompanied by an illustration of a male character, whereas the other was accompanied by an illustration of a female character. Both character illustrations were depicted as White. The profile included information about the character’s STEM interests and abilities. Each profile was designed to reflect equal STEM ability and interest. We initially pilot tested four profiles which focused on different STEM interests (computer science, astronomy, marine biology, cell biology) with 42 children (10–11-years-old) in the UK. The aim was to determine whether there were two profiles that children in this age range evaluated as of equal ability in science. Participants in this pilot study were asked how good the person described in the profile would be at science (1 = *not good at all*, 7 = *really good*). Paired sample *t*-tests were conducted to compare participants’ perceptions of the abilities of different profiles. The comparison between the astronomy (*M* = 5.54, *SD* = 1.50) and computer science (*M* = 5.83, *SD* = 1.01) profiles was non-significant (*t*(40) = 1.29, *p* = .21), suggesting that children in this age range rated these profiles as having approximately equal science ability. To further control for possible biases towards one of these domains, we counterbalanced whether the male or female character was paired with each profile.

**Table 1:** Peer Selection Task Character Profiles

Profile One (Astronomy)	Profile Two (Computer Science)
• Has been a science club member for three years	• Has been a science club member for three years
• Is really interested in learning about planets	• Is really interested in learning about robotics
• Would like to work in a planetarium	• Would like to work with computers

After reading about the science group scenario and the two profiles, participants were asked, “Who would you pick to help you with this difficult science question?” (male character, female character).

## Character Ability Perceptions

We also asked a single item question for each character “how good do you think this person usually is at science?” (1 = *really not good*, 6 = *really good*). As with the gender stereotype measure, we created a difference score (hereon, “male character ability perceptions”) by subtracting the female character ability score from the male character ability score. This was scaled from − 6 (minimum male-character ability favoring response) to + 6 (maximum male-character ability favoring response).

## Data Analysis Plan

The datasets used for the current study are not publicly available due to the ongoing nature of the research project but are available from the corresponding author on reasonable request.

To account for the multi-site nature of our data we calculated intra-class correlation coefficients (ICC) for our sites and exhibits within sites. For both site (ICC = 0.20) and exhibit within site (ICC = 0.23), the ICC for total stereotype endorsement suggested that multi-level modeling was the most appropriate analytic approach to account for the nested nature of our data. Thus, multilevel models were fit using the mixed command in SPSS Version 25 (IBM Corp, 2018) following best practices for multilevel modeling in SPSS (O’Dwyer & Parker, 2014) in order to account for variance based on exhibit and site.

Specifically, we carried out a 3 (Participant Age: Middle Childhood, Late Childhood, Adolescence) x 2 (Participant Gender: Girl, Boy) x 4 (STEM Domain: Science, Technology, Engineering, Mathematics) mixed model multivariate ANOVA. This allowed us to directly compare across the STEM domains to observe where differences in perceptions about male and female ability might lie based on participant age and gender. To break down interaction effects, pairwise comparisons were conducted, with Bonferroni corrections for multiple comparisons applied.

To supplement these analyses, we also carried out one-sample *t*-tests that compared the mean in a given condition (for example, boys in middle childhood) against the criterion value of 0 (i.e., the participant did not differ in their estimates of male and female ability). These *t*-tests allow us to determine whether participants were endorsing a stereotype or reporting no difference between male and female ability. The results of these *t*-tests are reported in full in Tables 2, 3, 4 and 5.

**Table 2:** Mean Stereotype Response and One-sample *t*-tests for Science as a Function of Participant Age and Gender

Age Group	Gender	*M*	*SD*	*t*	df	*p*	Cohen’s *d*
Middle Childhood	Boys	12.38	40.75	5.35	309	< 0.001	0.30
	Girls	-1.72	36.95	-0.83	316	0.41	0.05
Late Childhood	Boys	6.03	26.55	2.86	158	0.005	0.23
	Girls	-1.33	17.51	-1.02	180	0.31	0.08
Adolescence	Boys	0.27	16.61	0.15	85	0.88	0.02
	Girls	-1.46	15.11	-0.92	91	0.36	0.10

*Note*. For stereotype response scale; -100 = minimum stereotype endorsement, 0 = no difference between response to ‘boys’ and ‘girls’ questions, 100 = maximum stereotype endorsement.

**Table 3:** Mean Stereotype Response and One-Sample *t*-tests for Technology as a Function of Participant Age and Gender

Age Group	Gender	*M*	*SD*	*t*	df	*p*	Cohen’s *d*
Middle Childhood	Boys	12.61	40.56	5.49	310	< 0.001	0.31
	Girls	2.17	33.14	1.17	317	0.24	0.07
Late Childhood	Boys	13.27	32.68	5.09	156	0.005	0.41
	Girls	1.46	23.14	0.85	180	0.40	0.06
Adolescence	Boys	11.80	24.66	4.47	86	< 0.001	0.48
	Girls	6.66	20.71	3.10	92	0.003	0.32

*Note*. For stereotype response scale; -100 = minimum stereotype endorsement, 0 = no difference between response to ‘boys’ and ‘girls’ questions, 100 = maximum stereotype endorsement.

**Table 4:** Mean Stereotype Response and One-Sample *t*-tests for Engineering as a Function of Participant Age and Gender

Age Group	Gender		*M*	*SD*	*t*	df	*p*	Cohen’s *d*
Middle Childhood		Boys	27.35	44.12	10.92	309	< 0.001	0.62
		Girls	16.55	41.93	7.04	317	< 0.001	0.39
Late Childhood		Boys	18.48	34.68	6.66	155	< 0.001	0.53
		Girls	13.98	28.05	6.70	180	< 0.001	0.50
Adolescence		Boys	15.67	25.26	5.72	84	< 0.001	0.62
		Girls	11.37	24.88	4.36	90	< 0.001	0.46

*Note*. For stereotype response scale; -100 = minimum stereotype endorsement, 0 = no difference between response to ‘boys’ and ‘girls’ questions, 100 = maximum stereotype endorsement.

**Table 5:** Mean Stereotype Response and One-Sample *t*-tests for Math as a Function of Participant Age and Gender

	Gender	*M*	*SD*	*t*	df	*p*	Cohen’s *d*
Middle Childhood	Boys	6.37	36.37	3.08	309	0.002	0.18
	Girls	-5.05	35.40	-2.54	317	0.01	-0.14
Late Childhood	Boys	4.49	26.52	2.13	157	0.04	0.17
	Girls	-3.19	24.89	-1.73	180	0.09	-0.13
Adolescence	Boys	-3.62	17.14	-1.96	85	0.05	-0.21
	Girls	-0.48	19.01	-0.24	91	0.81	-0.03

*Note*. For stereotype response scale; -100 = minimum stereotype endorsement, 0 = no difference between response to ‘boys’ and ‘girls’ questions, 100 = maximum stereotype endorsement.

Finally, we conducted a binomial logistic regression that examined whether choice of male or female character in the peer selection task was predicted by participant gender, participant age, science stereotype endorsement and male character ability perceptions (within the peer selection task). We also included the two-way interaction term between participant age and participant gender.

## Results

### Multivariate Stereotype Analysis

The means and standard deviations for participants’ responses to the stereotype measures for both “boys” and “girls” questions are included as supplemental materials. The following analyses examine a stereotype response difference score as outlined in the methods section (where − 100 = minimum stereotype endorsement, + 100 = maximum stereotype endorsement). Examining stereotype responses across the four STEM domains in a multivariate ANOVA revealed significant multivariate main effects of participant gender (*F*(4, 1118) = 6.04, *p* < .001, *η*_*p*_*²* = 0.02) and participant age (*F*(4, 2238) = 2.18, *p* = .03, *η*_*p*_*²* = 0.008). The multivariate interaction between age group and gender was not significant (*F*(8, 2238) = 1.73, *p* = .087, *η*_*p*_*²* = 0.006). Tests of between-subjects effects within each domain follow.

## Science Stereotype

Examining science stereotype responses (see Table 2 for all means and standard deviations by participant age group and gender) revealed a significant main effect of gender, *F*(1, 1121) = 14.33, *p* < .001, *η*_*p*_*²* = 0.01. Boys reported significantly greater male stereotype endorsement for science than girls. This main effect was qualified by an interaction between participant gender and participant age group, *F*(2, 1121) = 3.40, *p* = .03, *η*_*p*_*²* = 0.006 (see Figs. 2 and 3). Pairwise comparisons revealed that within the middle childhood group, boys reported greater male stereotype endorsement for science than girls (*p* < .001). Similarly, in late childhood, boys reported greater male stereotype endorsement for science than girls (*p* = .03). By adolescence, there was no difference between the male stereotype endorsement for science reported by boys and girls (*p* = .71).

**Fig. 1:**
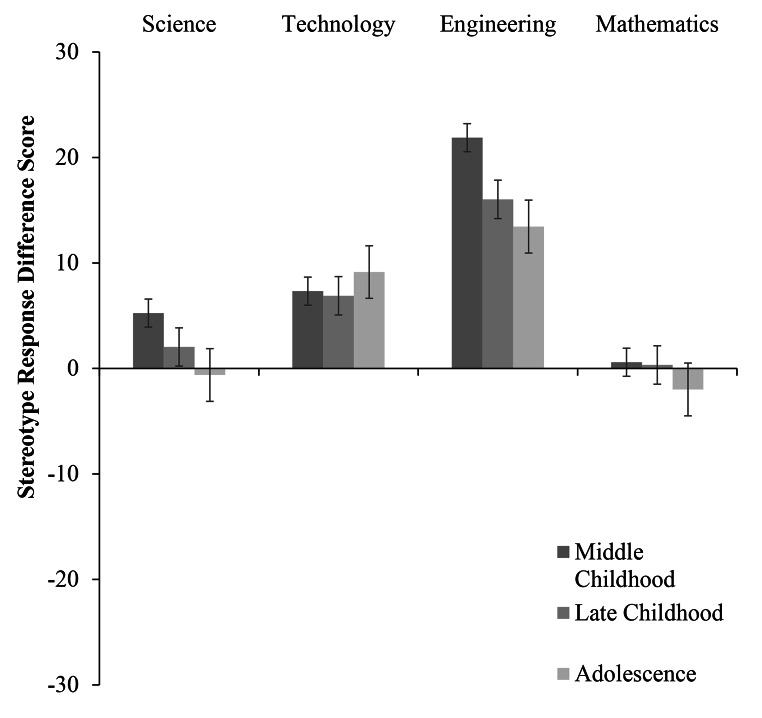
Stereotype Response as a Function of Participant Age and STEM Domain (w. Standard Error Bars)

**Fig. 2:**
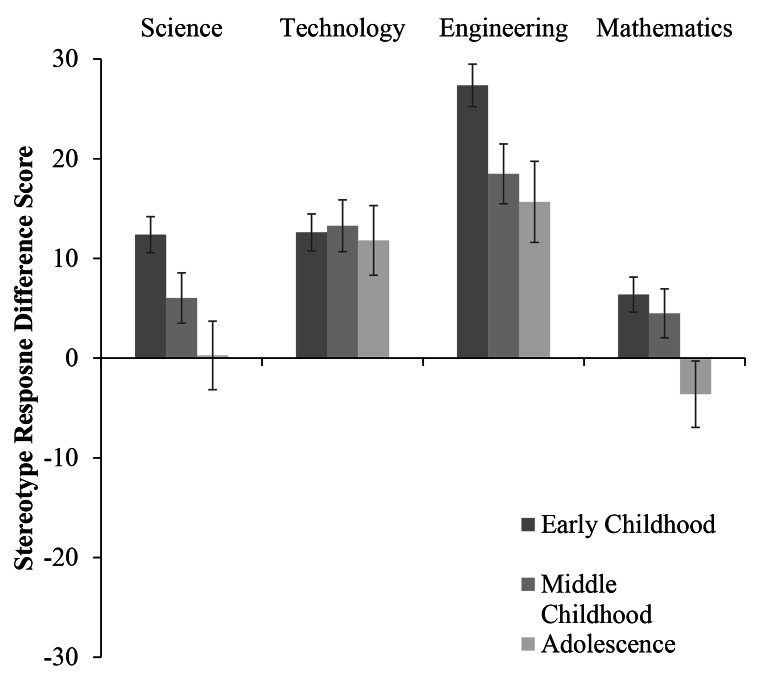
Stereotype Response as a Function of Participant Age for Male Participants (w. Standard Error Bars)

**Fig. 3:**
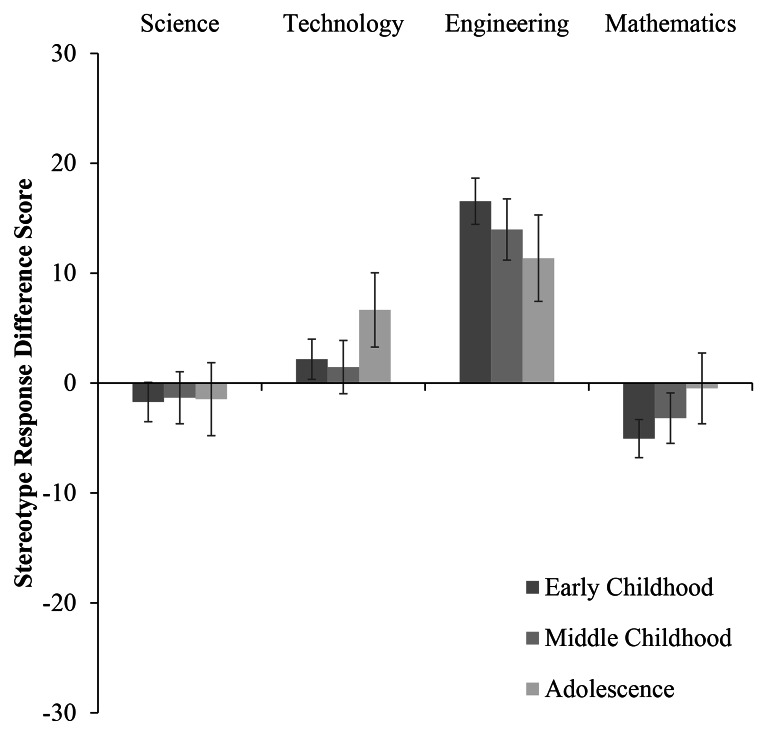
Stereotype Response as a Function of Participant Age for Female Participants (w. Standard Error Bars)

One-sample *t*-tests indicated that for boys in middle and late childhood, these responses were above the mid-point of the scale, while for girls in these age groups responses did not differ from the mid-point of the scale (see Table 2). Among girls, there were no differences in responses to the science stereotype measure between the age groups. Boys in adolescence reported significantly less male stereotype endorsement for science than boys in middle childhood (*p* = .003). There was no difference between boys’ responses in middle and late childhood, nor between boys’ responses in late childhood and adolescence.

## Technology Stereotype

Turning to technology stereotype responses (see Table 3 for all means and standard deviations by participant age group and gender), analyses revealed a significant main effect of gender, *F*(1, 1121) = 17.16, *p* < .001, *η*_*p*_*²* = 0.02. Overall, boys reported significantly greater male stereotype endorsement for technology than did girls. There were no main effects of participant age, nor did age and gender interact (see Table 3 for one-sample *t*-tests by age group and gender).

## Engineering Stereotype

When assessing engineering stereotype responses (see Table 4 for all means and standard deviations by participant age group and gender), we observed a main effect of gender, *F*(1, 1121) = 6.38, *p* = .01, *η*_*p*_*²* = 0.006. Boys reported significantly greater male stereotype endorsement for engineering than did girls. Further, there was a significant main effect of participant age group, *F*(2, 1121) = 5.12, *p* = .006, *η*_*p*_*²* = 0.009. Participants in middle childhood reported greater male stereotype endorsement for engineering than those in late childhood (*p* = .05) and adolescence (*p* = .02). There was no difference in male stereotype endorsement for engineering between participants in late childhood and adolescence (*p* = .99). Although male participants reported greater male stereotype endorsement, all participants across the three age groups displayed male stereotype endorsement for engineering (see Table 4 for one-sample *t*-tests by age group and gender).

## Mathematics Stereotype

Finally, examining math stereotype responses (see Table 5 for all means and standard deviations by participant age group and gender) revealed a main effect of gender *F*(1, 1121) = 5.49, *p* = .02, *η*_*p*_*²* = 0.005. Boys reported significantly greater male stereotype endorsement for math than did girls. In this case, girls’ responses were significantly below the mid-point of the scale, suggesting that overall, they responded in favor of girls’ math ability as opposed to boys’ ability (see Table 5 for one-sample *t*-tests by age group and gender). This effect was qualified by an interaction between participant age and gender, *F*(2, 1121) = 4.06, *p* = .02, *η*_*p*_*²* = 0.007 (see Figs. 2 and 3). In the middle childhood age group, boys reported greater male stereotype endorsement for math than girls did (*p* < .001). Similarly, in late childhood boys reported greater male stereotype endorsement for math than girls did (*p* = .02). In adolescence, there was no difference between boys’ and girls’ math stereotype measure responses (*p* = .38). Here, one-sample *t*-tests indicated that adolescent boys’ responses were below the mid-point of the scale and therefore in favor of female ability in mathematics. The only difference observed between age-groups was a significant difference between boys in middle childhood and boys in adolescence (*p* = .02), with greater endorsement of girls’ math ability in adolescence as compared to middle childhood.

## Peer Selection Task

Overall, 42% (*n* = 505) of participants selected the male character for help, whereas 53% (*n* = 637) of participants selected the female character for help. 5% of the sample did not complete this measure. Looking at this distribution based on participant gender, 63% (*n* = 390) of girls selected the female character, compared to 32% (*n* = 199) who selected the male character. In contrast, 53% (*n* = 306) of boys selected the male character, compared to 42% (*n* = 247) who selected the female character.

To examine the effect of stereotype responses on the peer selection task, we conducted a logistic regression model with character choice as the outcome (1 = choosing male character, see Table 6). Variance inflation factor (VIF) scores for each of our predictors (gender = 1.04, age = 1.01, science stereotype endorsement = 1.05, male character ability perception = 1.05) indicated that multicollinearity was not likely to cause issues in interpretation of the model. At step one, the model with main effects of participant age, gender, science stereotype endorsement, and male character ability perception was significant, *X*^*2*^(4) = 277.81, *p* < .001, Nagelkerke R^2^ = 0.31, and the non-significant Hosmer-Lemeshow test (*X*^*2*^(8) = 14.10, *p* = .08) indicated that the model fit the data well.

**Table 6:** Logistic Regression Analysis of Male Character Choice in Peer Selection Measure

Predictor	*Β*	SE *β*	Wald’s χ2	df	*P*	*eβ* (odds ratio)
Constant	-0.21	0.24	0.72	1	0.4	0.81
Gender (1 = female, 0 = male)	-0.69	0.14	23.53	1	0.001	0.50
Age	0.02	0.03	0.82	1	0.37	1.02
Science Stereotype Endorsement	0.01	0.002	6.15	1	0.01	1.01
Male Character Ability Perception	1.23	0.12	108.43	1	0.001	3.40

Among our predictors, male character selection was associated with being male (*β* = − 0.69, Wald *X*^*2*^(1) = 23.52, *p* < .001), greater male science stereotype endorsement (*β* = 0.01, Wald *X*^*2*^(1) = 6.15, *p* = .013), and greater male character ability perceptions (*β* = 1.25, Wald *X*^*2*^(1) = 112.90, *p* < .001). Participant age was not related to the likelihood of selecting the male character, *β* = 0.02, Wald *X*^*2*^(1) = 0.52, *p* = .47. At step two (*X*^*2*^(5) = 289.10, *p* < .001, Nagelkerke R^2^ = 0.32), the two-way interaction between participant age and gender was added to the regression model. This interaction term (*β* = 0.16, Wald *X*^*2*^(1) = 11.20, *p* < .001) indicated that counter to H3, for girls, there was an increased likelihood of selecting the male character to ask for help among the older girls compared to the younger girls.

## Discussion

The findings of the present study demonstrate that children and adolescents endorse different gender stereotypes about ability in the STEM domains. In partial support of H1, in the domains of science and math, boys in middle childhood endorsed the ability of their own gender group, compared to participants in late childhood and adolescence. In contrast to H1, there were no age differences among girls’ stereotypes in the science and math domains. Counter to H2, the greatest male stereotype endorsement, for *both* boys and girls *across* age groups, was observed in the domain of engineering, followed by technology. These findings emphasise the important point that the study of gender stereotypes requires domain specificity. Although much valuable research to date has focused on STEM as a broad construct (McGuire, Mulvey, et al., 2020) or looked individually within domains (Cvencek et al., 2011; Master et al., 2016, 2021), the present findings suggest that there are areas where greater disparities exist in children’s and adolescents’ stereotypical beliefs about gendered ability.

Further, the present findings demonstrate that there are consequences of male stereotype endorsement. In support of H3, greater endorsement of the abilities of the male character and science stereotype endorsement in general were related to selecting a male character for help with a difficult science task. Overall, girls’ selection of a male character was low across age groups. However, older girls were *more* likely than younger girls to select a male character for help, despite weaker stereotype endorsement among these older girls in the study. Such decisions may lead youth to reinforce ability stereotypes by asking boys for help in STEM contexts.

Examining stereotype responses across the STEM domains, we observed greater male stereotype endorsement in the domains of engineering and technology than in science or mathematics. Children’s and adolescents’ responses in the domains of engineering and technology reflect unequal gender representation and inequity in these areas (WISE, 2020b, 2021). In both engineering and technology, girls endorsed the stereotype of male ability. This meets with expectations derived from the social identity framework where members of lower status groups endorse stereotypes about the abilities of higher status groups. What is particularly troubling about this finding is that counter to our predictions, this endorsement appeared to emerge among participants in the youngest age group (here, as young as 5–8-years-old). In other research (McGuire, Mulvey, et al., 2020), girls in middle childhood did not endorse the idea that boys were inherently more able in STEM. However, with an examination of the individual domains, we see such endorsement of ability differences in this age range, mapping on to recent findings in interest stereotypes (Master et al., 2021). Here the societal transmission of these ideas (Cheryan et al., 2009, 2015) and observations of greater inequity in representation leading to early perceived status differences appear more powerful than the desire to maintain a positive view of one’s group.

Between middle and late childhood, we observed differences between the relative male stereotype endorsement reported in the engineering domain, but even among adolescents, responses still showed male stereotype endorsement among boys and girls. Similarly, in the technology domain, both boys and girls reported that boys were usually good at technology. This evidence further emphasises the importance of specificity in the study of STEM gender stereotypes. Here, broader inequity in engineering and technology (WISE, 2020b, 2021) is reflected in children’s responses to a stereotype measure where both girls and boys favor male ability. In contrast, in areas where women are more broadly represented and children have experience of girls’ achievement (i.e., observing girls succeed in the science and math classroom), male stereotype endorsement is smaller or reversed between middle childhood and adolescence.

In the domains of engineering and technology, children are likely to be exposed to a primarily male workforce (WISE, 2021). At the same time, in the traditional school curriculum in the US and the UK, children have fewer opportunities to engage with engineering and technology tasks, and therefore rely on observations of the workforce as a model to calibrate their expectations about gendered ability. This may help to explain our observed result where participants endorse boys’ ability in these areas, counter to evidence that demonstrates equal gendered ability in practice (Lindberg et al., 2010; Sullivan & Bers, 2013). Instead, this may provide further evidence for a self-sustaining cycle where stereotypes can reinforce under-representation. As demonstrated here, in childhood there is a belief, among both boys and girls, that boys are inherently more able in engineering and technology (see also Master et al., 2017). This view is reflected in the higher presence of men in these domains (WISE, 2020b), which likely serves to communicate these stereotypes to children in the first place, and further reinforce them once children endorse these ideas. This representation problem can be perpetuated by a loss of interest from girls who then do not pursue further study in these areas – which can, in part, be explained by gender stereotypes (Master et al., 2021).

In contrast, in the domain of mathematics, in middle childhood both boys and girls provided responses in favor of their own group’s math ability. The present paper extends the existing literature (e.g., Cvencek et al., 2011) by documenting adolescent boys reporting that girls’ abilities in math are greater than boys’ abilities. This aligns with meta-analytic findings that show no difference in ability or performance among boys and girls in math (Lindberg et al., 2010) as well as longitudinal data in the US that has shown girls closing the mathematics participation and achievement gap to outperform boys (Goldin et al., 2006). Our findings may reflect adolescent boys’ experiences in the classroom witnessing their female peers’ mathematical achievements.

In the domain of science, there was an age-related trend among boys who demonstrated in-group bias in middle childhood, before moving to respond more equitably in late childhood and adolescence. Girls, in contrast, did not endorse male stereotypes in this domain, nor did they show in-group bias. Aligned with work that has demonstrated a tendency for men to underestimate women’s STEM abilities (Grunspan et al., 2016), this finding suggests that the earliest demonstration of male stereotype endorsement in the broad domain of science can be observed among boys, not girls. Interestingly, this trend does not persist across age groups, even though boys remain part of a higher-status group, which again speaks to the possibility that exposure to the successes of their female peers in the classroom mitigates endorsement of ability stereotypes in science.

The present study also documents consequences for the science classroom related to stereotype endorsement. Participants, particularly girls, were more likely to select a girl for help with a difficult science task. One possibility is that this finding maps on to work from a stereotype content perspective that has demonstrated men are perceived to be more agentic (focused on themselves and achieving mastery, status and power), while women are perceived to be communal (focused on caring for others, displaying warmth and helping behaviors; Abele & Wojciszke 2014) and perhaps, therefore, better able to help in a classroom context. Alternatively, given the higher selection of the female character by girls (63% compared to 42% of boys), this may be more parsimoniously interpreted as an instance of in-group preference. Furthermore, older girls were *more* likely than younger girls to select a male character, despite science stereotype endorsement being weaker in late childhood and adolescence compared to middle childhood. This finding may reflect a decline in in-group bias or a desire to make egalitarian selections where boys are represented equally with girls. Research has documented that adolescents are concerned with issues of equity and equality (McGuire et al., 2019). Given this, future work would be beneficial to determine the *reasoning* that underlies children’s peer selection decisions. Asking children to report *why* they chose a certain peer will be an important next step to determine whether older girls chose a male character because they believed this person would be a more capable scientist, or whether they were concerned with equity.

Crucially, participants who showed greater male stereotype endorsement in their responses to the science stereotype measure were more likely to select a male character to ask for help with a difficult science question. These effects suggest that though we did see differences in male stereotype endorsement in the science domain between these age groups, when this endorsement persists, it can serve to perpetuate ideas about male dominance through more indirect pathways. Specifically, if children in the classroom need to seek help from their peers in a STEM context and ask a boy over a girl with equal ability and interest, this presents boys with the opportunity to vocalize their knowledge in this context and reinforce perceptions about boys’ abilities in the classroom. Again, we recognize some caution is required in interpreting these findings given that children may also be considering which peer they believe to be more empathic or well-organized (i.e., factors related to seeking help rather than STEM ability). However, we believe it is important to note that endorsement of male-favoring stereotypes can, in some cases, relate to decisions that may serve to uphold perceptions of superior male ability in the classroom.

The findings of the present study extend existing work in STEM gender stereotypes by demonstrating that these stereotypes are not unidimensional. Children and adolescents hold weaker stereotypes about gendered ability in math and science as compared to the engineering and technology domains. One possible explanation for this domain-based difference is that youth are more likely to encounter math and science than engineering and technology in their daily school lives, and accordingly have experiences of boys *and* girls succeeding. This could in turn lead to changes in *descriptive norms* about who can succeed in these areas. In general, boys and girls may note that currently men generally do technology and engineering jobs, and that boys more than girls are given toys that involve building and designing. Such examples set descriptive norms that children take into account when evaluating how acceptable it is for boys and girls to engage in different STEM domains (McGuire, Jefferys, et al., 2020).

Schools are beginning to incorporate more programming, coding, and robotics into their curricula (CAS, 2017). This offers promise as it will provide opportunities for students to observe their peers from different gender identity backgrounds succeeding in technology, which may in turn lead to changing descriptive norms about who is usually successful or skilled in these areas. An essential part of this shifting focus will be the recognition that boys and girls will enter these programs with existing stereotypes about their own gender groups. Research suggests that boys and girls are equally competent in early kindergarten robotics programs (Sullivan & Bers, 2013). Therefore, using early first-hand experiences of robotics and programming (e.g., Master et al., 2017) to challenge emergent technology and engineering stereotype endorsement will be an important means to promote gender equity in these areas.

## Limitations & Future Directions

In the present work, we used a direct explicit gender stereotype measure adapted from Liben & Bigler (2002). First, this measure uses single-items and as such replicating this work using scale measures should be a priority for future research. Further, some related work has used implicit association tests and other indirect measures to probe these stereotypes (Cvencek et al., 2011). It is possible that the more equitable responses to the direct stereotype measures used here involve some degree of self-presentation, which children in this age range have been shown to engage in (Fitzroy & Rutland, 2010). Future work should seek to complement and extend the present findings by examining implicit associations between gender and the different STEM domains. Further, the measure used relied on single items to ensure this was accessible for our youngest participants. Unfortunately, this meant we were unable to test for the reliability of this measure. Future work should examine explicit STEM gender stereotypes using multi-item measures where possible.

Similarly, future work is needed to extend our indirect peer selection task beyond the domain of science. Our findings demonstrate that gender stereotypes differ by STEM domain. It is quite possible that there would be different relationships between stereotypes and selection of a peer in the domains of math, engineering, and technology. In the latter two cases where there is a strong association with male ability for example, it is possible that participant gender would not predict selection of the male character, as both boys and girls endorsed ability stereotypes in these domains. Alternatively, older boys may be more likely to select a female character for help in the math domain. Understanding these domain-related differences will again help to inform the means to challenge these instances of bias in formal and informal educational settings.

Finally, the present work would benefit from a more inclusive and intersectional examination of how gender stereotypes may differ based on gender identity, ethnicity and socio-economic status. Here, participants could select from the gender identity options of ‘boy’, ‘girl’ or ‘other’. All of our participants selected either ‘boy’ or ‘girl’, but future work should seek to stratify sampling to include the representation of groups such as non-binary youth in order to examine perceptions of gender stereotypes outside of the gender binary sample. Further, researchers have demonstrated differences in stereotypes about STEM ability across gender and ethnic groups (O’Brien et al., 2015). The sample in the present work was composed of predominantly White British or White European American children and thus it is hard to say how reflective their responses to gender stereotype measures are of ideas about gendered ability in other ethnic groups (Master et al., 2021; Rowley et al., 2007). Therefore, future work should aim to examine the role of participant ethnicity in STEM gender stereotypes. Similarly, our participants were visitors to informal science learning sites. It is currently unclear whether children whose parents have the capital to invest in these kinds of informal science learning activities (often from middle to high socioeconomic status backgrounds) hold different STEM gender stereotypes, compared to children whose parents have less access to this capital. Therefore, future work should examine the relation between SES, science capital and STEM gender stereotypes among a more stratified sample recruited outside of informal science learning sites.

## Practice Implications

The findings of the present study document domain-related differences in children’s and adolescents’ endorsement of stereotypes. Within engineering and technology, there is an emerging belief that boys have a greater ability than girls. Aligned with a rapidly disappearing performance gap in math between boys and girls (Goldin et al., 2006), participants in the present work indicated that girls’ abilities were equal to or greater than the math abilities of their male peers. Experiences with age-appropriate engineering and technology exercises in schools that allow students to observe their peers succeeding in these areas will play a crucial role both in reducing the enrolment and performance gap in these domains, but also in challenging these stereotypes. These stereotypes in turn have implications for who children and adolescents will seek help from in a STEM context. Educators may wish to consider building group work activities with this in mind, by assigning children of different genders to work together, and implementing rules that ensure boys’ voices are not always the first or only ones heard in discussions about difficult STEM topics.

## Conclusion

The present study documents differences in science, technology, engineering and mathematics gender stereotypes across childhood and adolescence in a domain-specific manner. These differences point to a need to challenge gender stereotypes about male ability in the domains of engineering and technology among children from as young as five-years-old. An essential next step is to understand why there are differences in stereotypes between these domains, and how we can further promote gender equity in those areas with the greatest disparity.
